# Association of Affect and Gait Performance in Dual-Task Walking in Non-Demented Older Adults

**DOI:** 10.1093/geroni/igaa057.920

**Published:** 2020-12-16

**Authors:** Deepan Guharajan, Roee Holtzer

**Affiliations:** Yeshiva University, Bronx, New York, United States

## Abstract

Aging populations are at increased risk to experience mobility disability, which is associated with falls, frailty, and mortality. Previous studies have not examined the concurrent associations of both positive and negative affect with gait velocity. We examined whether individual differences in positive and negative affect predicted dual-task performance decrements in velocity in a dual-task (DT) paradigm in non-demented older adults. We hypothesize that positive affect would be associated with lower DT costs, and negative affect would be associated with higher DT costs. Participants (N = 403; mean age, = 76.22 (6.55); females = 56%) completed the Positive and Negative Affect Schedule (PANAS) and a DT paradigm that involved three task conditions: Single-Task-Walk (STW), Alpha (cognitive interference requiring participants to recite alternate letters of the alphabet), and Dual-Task-Walk (DTW) requiring participant to perform the two single tasks concurrently. Gait velocity was assessed via an instrumented walkway. As expected, results of a linear mixed effects model (LME) showed a significant decline in gait velocity (cm/s) from STW to DTW (estimate = -11.79; 95%CI = -12.82 to -10.77). LME results further revealed that negative affect was associated with greater decline in gait velocity from STW to DTW (ie., worse DT cost) (estimate = -0.38; 95%CI = -0.73 to -0.03). Positive affect did not, however, predict DT costs in gait velocity (estimate = -0.09; 95%CI = -0.23 to 0.05). These findings suggest that increased negative affect interferes with the allocation of attentional resources to competing task demands inherent in the DT paradigm.

